# Gastrointestinal Involvement in Muckle-Wells Syndrome: A Systematic Review of Clinical Presentation, Diagnostic Patterns, and Therapeutic Response

**DOI:** 10.7759/cureus.84572

**Published:** 2025-05-21

**Authors:** Aliaa H Alkhazendar, FNU Soxi, Qasim Zia, Vanesha Kumari, Rameet Kumar, Samia Israr, Maria Javed, Shafaq Mushtaq

**Affiliations:** 1 Surgery, Islamic University of Gaza, Gaza, PSE; 2 Internal Medicine, Jersey City Medical Center, Jersey City, USA; 3 Internal Medicine, Ibn-e-Siena Hospital and Medical Research Institute, Multan, PAK; 4 Internal Medicine, Jinnah Postgraduate Medical Centre, Karachi, PAK; 5 Internal Medicine, Jeejal Medical Center, Larkana, PAK; 6 General Medicine, Hinchingbrooke Hospital, Peterborough, GBR; 7 Medicine, Medcare International Hospital, Gujranwala, PAK; 8 College of Medicine, National University of Medical Sciences, Rawalpindi, PAK; 9 Surgery, Liaquat National Hospital, Karachi, PAK

**Keywords:** abdominal pain, autoinflammatory disease, caps, cryopyrin-associated periodic syndromes, gastrointestinal symptoms, il-1 inhibitors, muckle-wells syndrome, systematic review

## Abstract

Muckle-Wells syndrome (MWS), a rare autoinflammatory disorder within the cryopyrin-associated periodic syndrome (CAPS) spectrum, is primarily characterized by recurrent fevers, urticarial rash, sensorineural hearing loss, and risk of amyloidosis. Although systemic manifestations are well-documented, gastrointestinal (GI) symptoms remain underrecognized and poorly described. This systematic review explores the prevalence, diagnostic relevance, and treatment response of GI manifestations in MWS. A structured search strategy was employed using major databases, and studies were included if they involved patients with genetically or clinically confirmed MWS and reported GI symptoms such as abdominal pain or oral ulcers. A total of three studies met the inclusion criteria, including two observational cohorts and one case report. Abdominal pain was noted in up to one-third of patients with childhood-onset disease and recurrently in a confirmed case. While IL-1 blockade with anakinra or canakinumab demonstrated overall systemic improvement, GI outcomes were not consistently reported. These findings suggest that gastrointestinal involvement, though infrequently highlighted, may be clinically significant and should be integrated into diagnostic and therapeutic frameworks for MWS.

## Introduction and background

Muckle-Wells syndrome (MWS) is a rare autoinflammatory disorder and part of the cryopyrin-associated periodic syndrome (CAPS), caused by mutations in the NLRP3 gene encoding cryopyrin, a key component of the inflammasome complex [1]. Characterized predominantly by episodic fever, urticarial rash, sensorineural hearing loss, arthralgia, and a risk of secondary amyloidosis, MWS is a lifelong condition marked by dysregulated IL-1β activity and systemic inflammation [2]. The clinical presentation of MWS varies widely in severity and onset, often overlapping with other autoimmune or autoinflammatory diseases, contributing to diagnostic delays. In recent years, targeted IL-1 blockade therapies such as anakinra and canakinumab have revolutionized disease control and improved long-term outcomes [3,4].

Although MWS is primarily known for systemic and rheumatological involvement, emerging evidence has suggested that gastrointestinal (GI) symptoms may also be part of its clinical spectrum. Manifestations such as recurrent abdominal pain, nausea, oral ulcers, and inflammatory bowel disease-like features have been reported anecdotally or as part of broader clinical presentations [5]. These symptoms are often overlooked or misattributed to other GI disorders, leading to underrecognition and undertreatment. Importantly, given the systemic nature of IL-1-mediated inflammation, it is biologically plausible that the GI tract may be affected, either directly or indirectly, through amyloid deposition or chronic inflammation [6]. Despite the potential significance of GI involvement in MWS, there is a notable lack of consolidated literature examining its frequency, pathophysiological basis, diagnostic challenges, and therapeutic responses. Most of the existing information is scattered across case reports and small cohort studies, limiting generalizability. To address this gap, our systematic review aims to critically appraise and synthesize the available clinical evidence on GI manifestations in MWS, evaluate diagnostic patterns, and assess the efficacy of current treatment strategies, particularly IL-1-targeted therapies, in managing these symptoms.

To guide this review, we framed the following research question using the PICO format [7]: In patients with MWS (population), what are the reported GI manifestations and their underlying mechanisms (intervention/exposure), compared to patients without such symptoms or standard systemic features (comparison), in terms of accurate diagnosis, symptom burden, and response to treatment, including IL-1 inhibitors (outcome)?

## Review

Materials and methods

Search Strategy

The search strategy for this systematic review was designed in accordance with the Preferred Reporting Items for Systematic Reviews and Meta-Analyses (PRISMA) guidelines [8] to ensure a transparent and replicable methodology. A comprehensive literature search was conducted using major databases, including PubMed and Google Scholar, focusing on studies that reported GI manifestations in patients with genetically or clinically confirmed MWS. Keywords and MeSH terms such as “Muckle-Wells Syndrome,” “gastrointestinal symptoms,” “abdominal pain,” “oral ulcers,” and “IL-1 inhibitors” were employed in various combinations to maximize retrieval sensitivity. Articles were screened by title and abstract, followed by full-text review for eligibility. Inclusion criteria targeted clinical studies, cohort analyses, and case reports that discussed GI features, diagnostic evaluation, or treatment outcomes in MWS. Non-English articles, reviews without primary data, and studies focusing solely on other CAPS phenotypes were excluded. The PRISMA flow diagram was used to illustrate the study selection process and ensure methodological rigor.

Eligibility Criteria

Studies were included if they involved human participants with a clinical or genetic diagnosis of MWS and reported GI manifestations such as abdominal pain, oral ulcers, or related symptoms. Eligible study types included prospective or retrospective cohort studies, case series, and individual case reports. Only articles published in English were considered. Exclusion criteria included review articles without primary data, editorials, conference abstracts without full-text availability, and studies that did not distinguish MWS from other CAPS phenotypes. Additionally, studies focusing solely on systemic or non-GI aspects of MWS without mention of GI involvement were excluded from the final analysis.

Data Extraction

Data from the selected studies were extracted manually by the reviewers using a standardized form designed for this review. Extracted information included study design, sample size, patient demographics, reported GI symptoms, diagnostic methods, and clinical outcomes related to both GI and systemic features. For observational studies and cohorts, additional data such as use of IL-1 inhibitors and phenotypic classification were recorded when available. In the case of individual case reports, detailed clinical presentation and diagnostic confirmation were noted. Discrepancies in extracted data were resolved through discussion among reviewers to ensure accuracy and consistency.

Data Analysis and Synthesis

Given the heterogeneity of study designs and the limited number of available studies, a narrative synthesis was conducted in place of a quantitative meta-analysis. The analysis focused on identifying common patterns in GI symptom presentation, subgroup characteristics (e.g., early-onset inflammatory phenotype), diagnostic approaches, and therapeutic outcomes. Special attention was given to whether IL-1 blockade was associated with improvement in GI manifestations. The synthesized data were tabulated to facilitate cross-study comparisons and to highlight key clinical features. Risk of bias and study quality were assessed using appropriate tools for observational studies and case reports to contextualize the strength of the evidence.

Results

Study Selection Process

**Figure 1:**
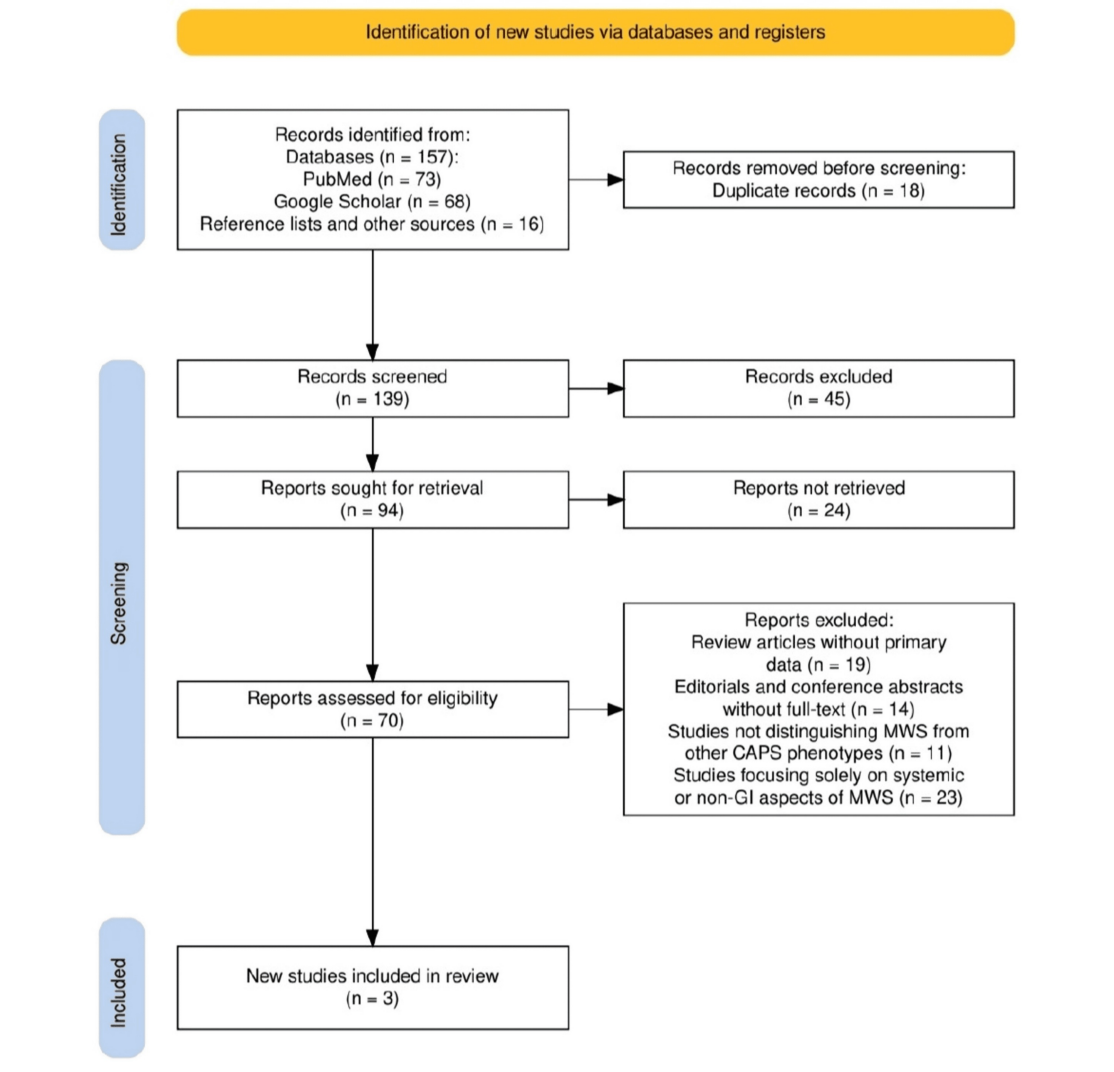
The PRISMA flowchart represents the study selection process PRISMA: Preferred Reporting Items for Systematic Reviews and Meta-Analyses; CAPS: cryopyrin-associated periodic syndrome; MWS: Muckle-Wells syndrome; GI: gastrointestinal

The study selection process, illustrated in Figure 1, was conducted in accordance with the PRISMA 2020 guidelines to ensure a transparent and structured approach. An initial total of 157 records were identified: 73 from PubMed, 68 from Google Scholar, and 16 from reference lists and other sources. After removing 18 duplicate entries, 139 titles and abstracts were screened for relevance. Of these, 45 were excluded for not meeting the inclusion criteria. Seventy full-text articles were then assessed for eligibility, while 24 could not be retrieved. Out of the 67 full-text articles looked at, 19 were left out because they didn't have the main data needed, 14 were just editorials or conference summaries without full text, 11 didn't separate MWS from other CAPS types, and 23 only talked about non-GI parts of MWS. As shown in Figure 1, this process ultimately led to the inclusion of three studies in the final systematic synthesis.

Characteristics of the Selected Studies

The key characteristics of the included studies are outlined in Table 1, reflecting a mix of observational and descriptive research that highlights the limited but notable evidence on GI manifestations in MWS. The studies varied in design, including two cohort studies and one case report, with sample sizes ranging from a single patient to over thirty participants. Patient demographics spanned a broad age range, with both pediatric and adult cases represented, and all had confirmed NLRP3 mutations. GI symptoms such as abdominal pain and oral ulcers were reported across the studies, with one cohort identifying abdominal pain in 31% of childhood-onset cases, suggesting a potential link to the inflammatory phenotype. While all studies employed clinical and genetic diagnostic methods, GI symptoms were not consistently emphasized as outcome measures. As shown in Table 1, most outcomes focused on systemic disease activity and treatment response, highlighting the need for more targeted investigation into the GI aspects of MWS.

**Table 1:** Characteristics of the included studies in the review MWS-DAS: Muckle-Wells Syndrome-Disease Activity Score; CRP: C-reactive protein; ESR: erythrocyte sedimentation rate; SAA: serum amyloid A; S100A12: S100 calcium-binding protein A12; GI: gastrointestinal; NLRP3: NACHT, LRR, and PYD domains-containing protein 3 (also known as cryopyrin)

Study (Author, Year)	Study Design	Sample Size	Patient Demographics	Reported GI Manifestations	Diagnostic Methods	Outcome (GI + Systemic)
Kuemmerle-Deschner et al., 2013 [9]	Prospective observational cohort	26 (12 Anakinra, 14 Canakinumab)	Mixed age (3–72 yrs), 57% female, NLRP3 mutations	Abdominal pain and oral ulcers are included in MWS-DAS but not emphasized in outcomes	Genetic testing, MWS-DAS, CRP, ESR, SAA, S100A12	Systemic improvement noted; GI outcomes not specifically reported
Kuemmerle-Deschner et al., 2014 [10]	Multicenter cohort study	34 patients	Mixed age (0.5–75 yrs), 47% male, genetically confirmed MWS	Abdominal pain in 31% of childhood-onset group; part of “inflammatory phenotype”	Clinical history, genetic confirmation, correspondence analysis	Phenotypic classification: GI symptoms are more frequent in early-onset MWS
Koike et al., 2007 [11]	Case report	1 patient	23-year-old female	Recurrent abdominal pain during inflammatory episodes	Clinical symptoms and H312P NALP3 mutation testing	GI symptoms noted during classic MWS flares; diagnosis based on clinical-genetic match

Quality Assessment

The quality assessment of the included studies is summarized in Table 2, using appropriate tools based on study design. Both cohort studies were evaluated with the National Institutes of Health Quality Assessment Tool for Observational Cohort Studies [12] and were rated as having moderate overall quality. While these studies were not randomized, the use of consecutive patient enrollment helped reduce selection bias in one, whereas the other exhibited some potential bias due to recruitment from specialized centers. Data completeness was generally strong, with detailed clinical and laboratory findings, although longitudinal follow-up was more robust in the prospective cohort than the cross-sectional study. Outcome measures, particularly related to systemic disease activity, were clearly defined; however, GI outcomes were inconsistently emphasized or underreported. The case report was appraised using the Joanna Briggs Institute (JBI) Critical Appraisal Checklist [13] and rated as low to moderate quality. Despite its single-patient focus, it provided a comprehensive clinical description, including well-documented GI symptoms within the broader MWS context. Overall, the quality of evidence highlights the need for more rigorous and GI-focused studies in this area.

**Table 2:** The quality assessment of the included studies in the review NIH: National Institutes of Health; JBI: Joanna Briggs Institute; MWS-DAS: Muckle-Wells Syndrome Disease Activity Score; GI: gastrointestinal

Study (Author, Year)	Study Design	Tool Used	Randomization/Selection Bias	Completeness of Data	Clarity of Outcome Measures	Overall Quality Judgment
Kuemmerle-Deschner et al., 2013 [9]	Observational cohort	NIH Quality Assessment Tool for Observational Cohort Studies	Not randomized; consecutive patient enrollment reduces bias	Complete clinical and lab data reported	Clear outcomes (MWS-DAS, inflammatory markers); GI outcomes underreported	Moderate
Kuemmerle-Deschner et al., 2014 [10]	Multicenter cohort study	NIH Quality Assessment Tool for Observational Cohort Studies	No randomization; potential selection bias due to recruitment centers	Cross-sectional; some clinical variables were not followed over time	Phenotypic classification is clear; GI outcomes are not primary	Moderate
Koike et al., 2007 [11]	Case report	JBI Critical Appraisal Checklist for Case Reports	Not applicable (single patient)	Complete description of case presentation	GI symptoms are clearly stated; systemic context is explained	Low to moderate (single case but relevant and clearly reported)

Discussion

This systematic review identified GI manifestations in MWS as underrecognized but potentially relevant clinical features, especially in specific patient subgroups. Across the three included studies, abdominal pain emerged as the most consistently reported GI symptom, with oral ulcers noted as a secondary feature. In the prospective cohort by Kuemmerle-Deschner et al. [9], GI symptoms such as abdominal pain and oral ulcers were included within the MWS Disease Activity Score (MWS-DAS), although they were not emphasized in outcome reporting or analyzed independently. In contrast, their 2014 multicenter cohort study [10] highlighted a notable prevalence of abdominal pain, reported in 31% of patients diagnosed in childhood, suggesting a predilection for GI involvement within the “inflammatory phenotype” associated with early-onset disease. This supports the notion that GI symptoms may be more prominent in younger MWS patients or those with a more systemic inflammatory presentation. The case report by Koike et al. [11] further reinforces this association, documenting recurrent abdominal pain as part of the patient’s inflammatory episodes. While all studies primarily focused on systemic disease control, those involving IL-1 blockade (anakinra or canakinumab) reported overall clinical improvement; however, none directly assessed the resolution of GI symptoms. These findings collectively indicate that while GI involvement in MWS may be overlooked in larger studies, it is clinically present in a subset of patients, particularly children, and may respond to anti-IL-1 therapy, although targeted evidence remains limited.

The pathophysiology of GI involvement in MWS is not well-defined but can be plausibly explained by the disease’s underlying immunologic mechanisms. MWS is driven by gain-of-function mutations in the NLRP3 gene, leading to excessive activation of the NLRP3 inflammasome and overproduction of IL-1β, a potent pro-inflammatory cytokine [14]. Elevated IL-1β levels can cause systemic inflammation affecting multiple organs, including the GI tract. Chronic inflammation may lead to mucosal damage, altered gut motility, or nonspecific abdominal pain [15]. In advanced cases, persistent inflammation and elevated serum amyloid A (SAA) can result in secondary amyloidosis, a known complication in MWS that can involve the kidneys and, potentially, the intestines, causing abdominal discomfort, diarrhea, or malabsorption [16]. Though rarely discussed explicitly, this immunopathological basis supports the biological plausibility of GI manifestations in MWS.

Clinically, GI symptoms in MWS may be underrecognized, contributing to delayed or missed diagnoses. This is particularly problematic given the nonspecific nature of symptoms such as abdominal pain and oral ulcers, which may be misattributed to unrelated GI or autoimmune conditions. The MWS-DAS [17,18] does incorporate abdominal pain and oral ulcers among its evaluated domains, but these components are seldom emphasized in clinical outcomes or treatment monitoring, leading to their diagnostic underappreciation. Importantly, early genetic testing for NLRP3 mutations in patients with recurrent, unexplained inflammatory episodes, including those with GI complaints, could facilitate earlier recognition of MWS [19] and reduce diagnostic latency, especially in pediatric cases or those with atypical presentations.

From a therapeutic standpoint, IL-1 inhibitors such as anakinra and canakinumab are well established in achieving systemic control in MWS [20,21], as demonstrated by consistent reductions in inflammatory markers and disease activity scores across studies [22]. However, specific data regarding the resolution of GI symptoms with IL-1 blockade remain limited. While none of the reviewed studies measured GI outcomes as primary endpoints, the observed systemic remission suggests that GI symptoms driven by the same inflammatory mechanisms may also respond to targeted therapy. Anecdotal reports, such as those from the included case study, support this hypothesis. These findings imply that GI manifestations in MWS could improve in parallel with systemic disease when IL-1-mediated inflammation is effectively suppressed [23].

The included studies in this review were limited by small sample sizes, observational or descriptive designs, and a lack of focus on GI outcomes. None of the studies used standardized GI symptom scales or explicitly measured changes in GI manifestations following treatment. Additionally, the inclusion of a single case report, while valuable in rare disease research, further limits the generalizability of findings. The heterogeneity in patient populations, treatment regimens, and outcome reporting complicates efforts to draw firm conclusions about the frequency, severity, or treatment responsiveness of GI symptoms in MWS.

Future research should prioritize larger, prospective cohort studies that specifically monitor GI symptoms alongside systemic disease activity in MWS. Standardized GI symptom documentation should be integrated into CAPS disease activity tools and treatment trials [24]. Furthermore, future IL-1 inhibitor studies should include GI outcomes as part of their efficacy assessments to better understand how anti-IL-1 therapy impacts these potentially overlooked manifestations. Such efforts will help clarify the true burden of GI involvement in MWS and improve comprehensive disease management.

## Conclusions

GI manifestations in MWS, though infrequently reported and often underexplored, may represent a clinically meaningful aspect of the disease spectrum, particularly in early-onset or inflammatory phenotypes. This review highlights the need for increased clinician awareness of abdominal pain and oral ulcers as possible components of MWS, which may aid in earlier diagnosis and more holistic management. By systematically examining available evidence, this study addresses a notable gap in the literature and underscores the importance of incorporating GI symptom assessment into future clinical evaluations and research on MWS. These findings provide a foundation for more targeted investigations and the development of standardized approaches to recognizing and managing GI involvement in this rare autoinflammatory disorder.
